# Chronic Sheehan’s Syndrome – A Differential to be Considered in Clinical Practice in Women with a History of Postpartum Hemorrhage

**DOI:** 10.7759/cureus.6290

**Published:** 2019-12-04

**Authors:** Merin Jose, Saba Amir, Rajesh Desai

**Affiliations:** 1 Internal Medicine, Saint Peters University Hospital, New Brunswick, USA

**Keywords:** case report, panhypopituitarism, central hypothyroidism, delay in diagnosis, amenorrhea, chronic, fatigue, hypothyroidism, adrenal insufficiency, sheehan's syndrome

## Abstract

Sheehan's syndrome is hypopituitarism due to pituitary gland necrosis resulting from hemorrhagic shock during pregnancy. It is a rare complication with varied manifestations and a considerable delay in diagnosis. We describe the case of a 36-year-old female with eight years of non-specific symptoms of generalized myalgias and intense fatigue managed symptomatically all these years. Further clinical assessment revealed amenorrhea and agalactia ongoing for several years without a clinical diagnosis. A good history and physical led to the diagnosis during a routine outpatient visit. She had significant improvement noted following the commencement of treatment. Previous case reports describe cases being diagnosed after one or other complications from long-term panhypopituitarism. Through this case, we want to illustrate that undiagnosed Sheehan's syndrome is associated with long-term morbidity, and there should be a high index of suspicion for it to be diagnosed during a routine clinical visit and thus prevent complications before a diagnosis can be made. It is essential to create awareness, especially in developed countries like the United States, where it has received less attention over the last few years.

## Introduction

Sheehan's syndrome is hypopituitarism due to postpartum ischemic necrosis of the pituitary gland [1]. Pregnant women are at higher risk of ischemic necrosis of the pituitary gland due to several factors - a) doubling of the pituitary gland size as a physiological response during pregnancy which, in turn, can compress the blood vessels around it [1], and b) a high risk of hemorrhagic shock during delivery if appropriate measures are not taken and sometimes even with the adequate measures. The prevalence has been coming down over the years with better diagnostic and treatment modalities. In a study in Iceland, the prevalence was 5.1 per 100,000 which was higher than the expected for a developed country [2]. The prevalence is much higher in developing countries and has been noted to be as high as 3.1% in a state in India where more than half of the affected individuals had home deliveries [3]. 

A study in France showed that the delay in diagnosis of Sheehan's syndrome was 9 ± 9.7 years [4] and a longer delay of 20.37  ±  8.34 years noted in developing countries [5]. Previous case reports have depicted Sheehan's syndrome being diagnosed after presenting with altered mental status from complications, such as recurrent hypoglycemia and severe hyponatremia [6-7]. Hemorrhagic shock during pregnancy is a key leading point in diagnosis. The objective of our case report is to emphasize the need to consider chronic Sheehan's syndrome in patients with a history of postpartum hemorrhage who present with non-specific or subtle changes during routine clinical visits and to have a high index of suspicion to avoid the delay in diagnosis and prevent complications of a rare but treatable condition.

## Case presentation

A 36-year-old female presented to our clinic for the first time with chronic pain in bilateral upper and lower extremities (predominantly in the proximal part) and generalized fatigue and lethargy for the past eight years, insidious in onset and gradually progressing. The pain and fatigue worsened over the years to the extent that it stopped her from going to work. At a baseline, she could carry out her day-to-day activities and came to the clinic by herself. 

On further evaluation, it was noted that her movements were sluggish, she had slow speech with a hoarse voice, dry and thick skin, and mentioned difficulty remembering things. The Patient Health Questionnaire-9 (PHQ-9) score was suggestive of severe depression.

The only significant medical history that she knew and could remember was that following her last childbirth eight years earlier, she lost consciousness and had profound vaginal bleeding following which a hysterectomy was done and she had been sick since then. She had five pregnancies and breastfed all her four children, except her last child whom she could not as she did not have any breast milk despite trying several remedies. As per the patient, she visited several doctors in the last several years for the same and was given symptom-driven treatment with no long-term benefits. She was unsure about the medications she had received and the workup that was done. Previous records from her providers could not be obtained as she did not have a single established provider.

**Figure 1:**
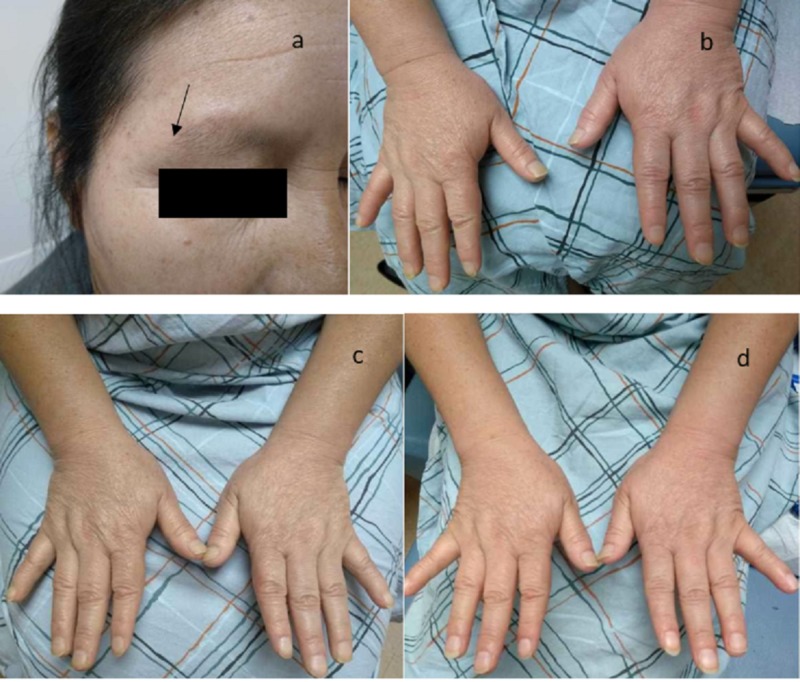
Clinical findings on presentation and changes with treatment a) loss of lateral one-third of the eyebrows at the time of diagnosis; b) hands at the time of diagnosis; c) slight decrease in the puffiness of the hands after two weeks of treatment; d) hands after six weeks of treatment

The examination was significant for a slow response to commands, facial puffiness, and loss of a lateral third of the eyebrows (Figure 1). The skin was cold, thick, and dry. Blood pressure was 80-90/50-70 mmHg. She was orthostatic positive with a postural fall in diastolic blood pressure of > 10 mmHg. She had severe tenderness of the proximal muscles in the upper and lower extremities bilaterally. No localized joint tenderness or limitation in the range of motion of joints was noted. 

**Figure 2:**
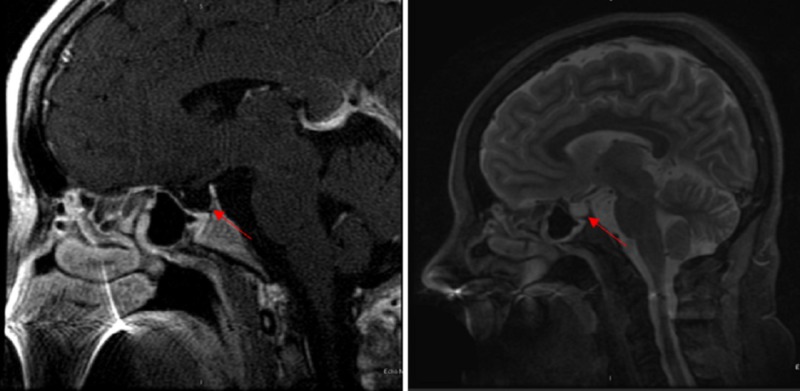
Magnetic resonance imaging (MRI) of the brain showing an empty sella

Based on her history, a working diagnosis of hypothyroidism with a concern for central hypothyroidism, possibly secondary to Sheehan's syndrome, was made. All the labs were as listed in Table 1 which was suggestive of panhypopituitarism. Given the very low serum cortisol level and multiple hormone deficiencies, an adrenocorticotropic hormone (ACTH) stimulation test was not performed. Magnetic resonance imaging (MRI) of the brain showed an empty sella (Figure 2) confirming the diagnosis of Sheehan’s syndrome.

**Table 1:** Significant/Abnormal Laboratory Results Significant laboratory results (SN 1 - 8) confirming panhypopituitarism; SN 9 - 15: other abnormal and significant laboratory results of the patient L: lymphocytes; N: neutrophils

SN	Parameter	Patient’s blood level		Reference range
1	Thyroid-stimulating hormone		0.746 uIU/mL	0.465 - 4.68 uIU/mL
2	Triiodothyronine		0.56 ng/mL	0.97 - 1.69 ng/mL
3	Tetraiodothyronine or thyroxine		< 0.25 ng/dL	0.79 - 2.35 ng/dL
4	Cortisol		< 0.4 mcg/dL	4.5 - 22.7 mcg/dL
5	Luteinizing hormone		< 0.20 mIU/mL	0.5 - 76.3 mIU/mL
6	Follicular stimulating hormone		1.6 m IU/mL	2.5 - 116 IU/mL
7	Prolactin		< 1 ng/ml	3.0 - 30.0 ng/ml
8	Creatinine kinase		1,538 µ/L	30.0 - 135.0 µ/L
9	Somatomedin		< 16 ng/ ml ng/mL	53 - 331 ng/mL
10	White blood cell count (differential)	6.8 (N - 33%, L - 62%) x 10^3^/mm^3^		4.0 - 11.0 x 10^3^/mm^3^
11	Serum sodium	132 mmol/L		136 - 145 mmol/L
12	Alkaline phosphatase	144 µ/L		42 - 98 µ/L
13	Aspartate aminotransferase	108 µ/L		14 - 36 µ/L
14	Alanine aminotransferase	42 µ/L		9 - 52 µ/L
15	Total bilirubin	1.3 mg/dL		0.1 - 1.2 mg/dL
16	Vitamin D	10 ng/mL		25 - 80 ng/mL
17	C-reactive protein, rheumatoid factor, antinuclear antibodies	Normal		NA

Following the diagnosis, the patient was referred to an endocrinologist. Initially, she was started on hydrocortisone, 10 mg in the morning and 5 mg in the evening. One week following that, she was started on levothyroxine once daily and doses were adjusted based on her follow-up laboratory results as an outpatient by the endocrinologist. Following treatment, her myalgias resolved completely. Her puffiness decreased, which is depicted in Figure 1c-d. Her PHQ-9 score showed that she was not depressed anymore. Her speech improved, and the thickness of her skin also started improving.

## Discussion

In 1914, hypopituitarism was first described by Simmonds [8]. Almost 20 years later, Sheehan’s syndrome was described [9]. The incidence has been coming down, especially in developed countries, and has received less attention in the last few years [2]. However, a study in Iceland in 2009 showed a higher prevalence than expected [2].

The anterior pituitary is more vulnerable than the posterior [6]. In our patient, the clinical presentation and laboratory studies were suggestive of anterior pituitary involvement. It presents with varied manifestations. In a study from France analyzing 28 patients with Sheehan's syndrome, 50% presented with agalactia, 25% had headaches in the postpartum period, 27.3% presented with amenorrhea, 25% had hypothyroidism, and 42.8% had asthenia since delivery [4]. In another study from India (representing a developing country), analysis of 18 patients with Sheehan's syndrome showed that 39% presented following complications with hyponatremia, hypotension, hypoglycemia, or vomiting, 16.7% presented with asthenia and weight loss, 11.1% had hypothyroidism, 94.4% patients had lactational failure, and 72.2% had amenorrhea following the last delivery [10]. These varied presentations reflect the higher likelihood of missing the diagnosis unless a good history is obtained and a thorough physical examination is done.

Sheehan's syndrome can be acute or chronic [6]. Acute cases present with failure to lactate or amenorrhea. Our patient could not breastfeed following her pregnancy due to lactation failure, indicating an acute presentation during which she tried home remedies. In the study in France, the mean diagnostic delay for patients with agalactia was 2.52 ± 3 months and was 8.3 ± 8 years for patients with amenorrhea [4]. On top of that, over the years, our patient also developed signs and symptoms of chronic Sheehan's syndrome, including subtle features of secondary hypothyroidism progressing to hypothyroid myopathy and secondary adrenal insufficiency which was missed by multiple providers. The delay in diagnosis in patients presenting with hypothyroidism was 8.1 ± 8.5 years and those presenting with acute adrenal insufficiency was 10.6 ± 9.4 years [4]. In patients who present with acute disease progressing to chronic conditions, a diagnosis could have been made at several stages. In our patient, the first clue to her diagnosis was her lactational failure, the next clue was the manifestation of symptoms of hypothyroidism in a subtle way with asthenia and myalgia, and then it progressed to adrenal insufficiency with dizziness and hypotension, which were all missed as findings in making a diagnosis. Several factors would have contributed to it, such as the lack of awareness, especially given the fact that patients with panhypopituitarism can walk into the clinic just like any other patient with non-specific complaints, and the lack of a thorough history and physical required to diagnose a rare disease with such varied presentation.

Although hypothyroidism can be brought up as a differential based on the symptoms our patient presented with, it is essential to consider central hypothyroidism. In this case, however, although the thyroid-stimulating hormone (TSH) was normal, the consideration for central hypothyroidism prompted a further workup. The normal TSH would have been one of the reasons the diagnosis was missed for so many years, although she had nonspecific features of hypothyroidism.

Due to the delay in diagnosis, significant morbidity with consequences was noted with substantial psychological stress, including the loss of employment. Patients pay a heavy price when a correct and timely diagnosis is not made as noted in one of the cases reported in a developing country where the financial loss prior to diagnosis due to the delay in diagnosis was close to $10,000, and following the diagnosis, the expense was $14/month for the patient [7].

## Conclusions

Thus, although rare, there should be a high index of suspicion for Sheehan’s syndrome by primary care physicians in patients with an obstetric history of intrapartum or postpartum hemorrhage. It is associated with increased morbidity and mortality if not diagnosed early. Hence, through this case report, we emphasize the need for clinicians to look for the symptoms of hypopituitarism through a good history and physical examination. Physicians should also educate their patients, especially in the event of hemorrhage during pregnancy or delivery, of possible hypopituitarism in the near or long-term future and advise patients to seek medical attention in case of any concerning symptoms. Thus, creating awareness and a timely diagnosis can avoid the poor quality of life that can span for several years and prevent precipitating complications.
